# A case of undifferentiated pleomorphic sarcoma in esophagus after multiple cancer treatments of surgery and chemoradiotherapy

**DOI:** 10.1186/s40792-022-01560-0

**Published:** 2022-11-04

**Authors:** Yuho Ebata, Yoshihisa Sakaguchi, Yasuo Tsuda, Sho Nambara, Kensuke Kudou, Eiji Kusumoto, Rintaro Yoshida, Tetsuya Kusumoto, Koji Ikejiri

**Affiliations:** Department of Gastroenterological Surgery and Clinical Research Institute, Kyushu Medical Center, Jigyohama 1-8-1, Chuo-Ku, Fukuoka, 810-8563 Japan

**Keywords:** Undifferentiated pleomorphic sarcoma, Cancer treatment, Esophagectomy

## Abstract

**Background:**

Undifferentiated pleomorphic sarcoma (UPS) in the esophagus is extremely rare. Therefore, there are few reports of UPS in the esophagus (UPSE). We present a case of UPSE after multiple cancer treatments.

**Case presentation:**

A 73-year-old man with a history of cancer treatment, including distal gastrectomy, transverse colectomy, and chemoradiotherapy, was diagnosed with an elevated lesion such as a submucosal tumor in the lower esophagus by regular endoscopy. A boring biopsy was performed, and the specimen showed features of sarcoma. The patient underwent a partial esophagectomy without lymph node dissection. Histopathological findings confirmed an undifferentiated pleomorphic sarcoma. Adjuvant therapy was not administered, and the patient survived without recurrence 1 year after surgery.

**Conclusions:**

Currently, complete resection is the only treatment option for UPSE. An optimal treatment strategy using chemotherapy or radiotherapy should be established.

## Background

Undifferentiated pleomorphic sarcoma (UPS), also known as malignant fibrous histiocytoma (MFH), is the second most common type of tissue sarcoma [1]. It occurs in soft tissues, retroperitoneum, and sometimes in the digestive tract, such as the stomach or colon; however, the esophagus is extremely rare as a primary site [2]. Therefore, there are few reports of UPS in the esophagus (UPSE), and the etiology and epidemiology remain unclear.

We report a case of UPSE with a history of cancer treatment that was successfully treated by surgery and present a review of the current literature on UPSE.

## Case presentation

A 73-year-old man with a history of cancer treatment was referred to our department for further evaluation and treatment of an esophageal tumor, found on regular endoscopy. The patient underwent a distal gastrectomy (Billroth I reconstruction) for gastric cancer 29 years ago, transverse colectomy for colon cancer 12 years ago, endoscopic submucosal dissection (ESD) for remnant gastric cancer 11 years ago, ESD for upper thoracic esophageal cancer 10 years ago, endoscopic laryngopharyngeal surgery for laryngeal and hypopharyngeal cancer 7 years ago, chemoradiotherapy for recurrence of laryngeal cancer 4 years ago, ESD for upper thoracic esophageal cancer 2 years ago, and transoral laryngoscopic surgery for hypopharyngeal cancer 1 year ago. Some medications were prescribed to manage comorbidities including type 2 diabetes, hyperlipidemia, hypothyroidism, and prostatic hypertrophy. The patient was an ex-smoker (daily 30 cigarettes until 60 years) and ex-drinker (daily 6 bottles of beer until 66 years).

**Fig. 1:**
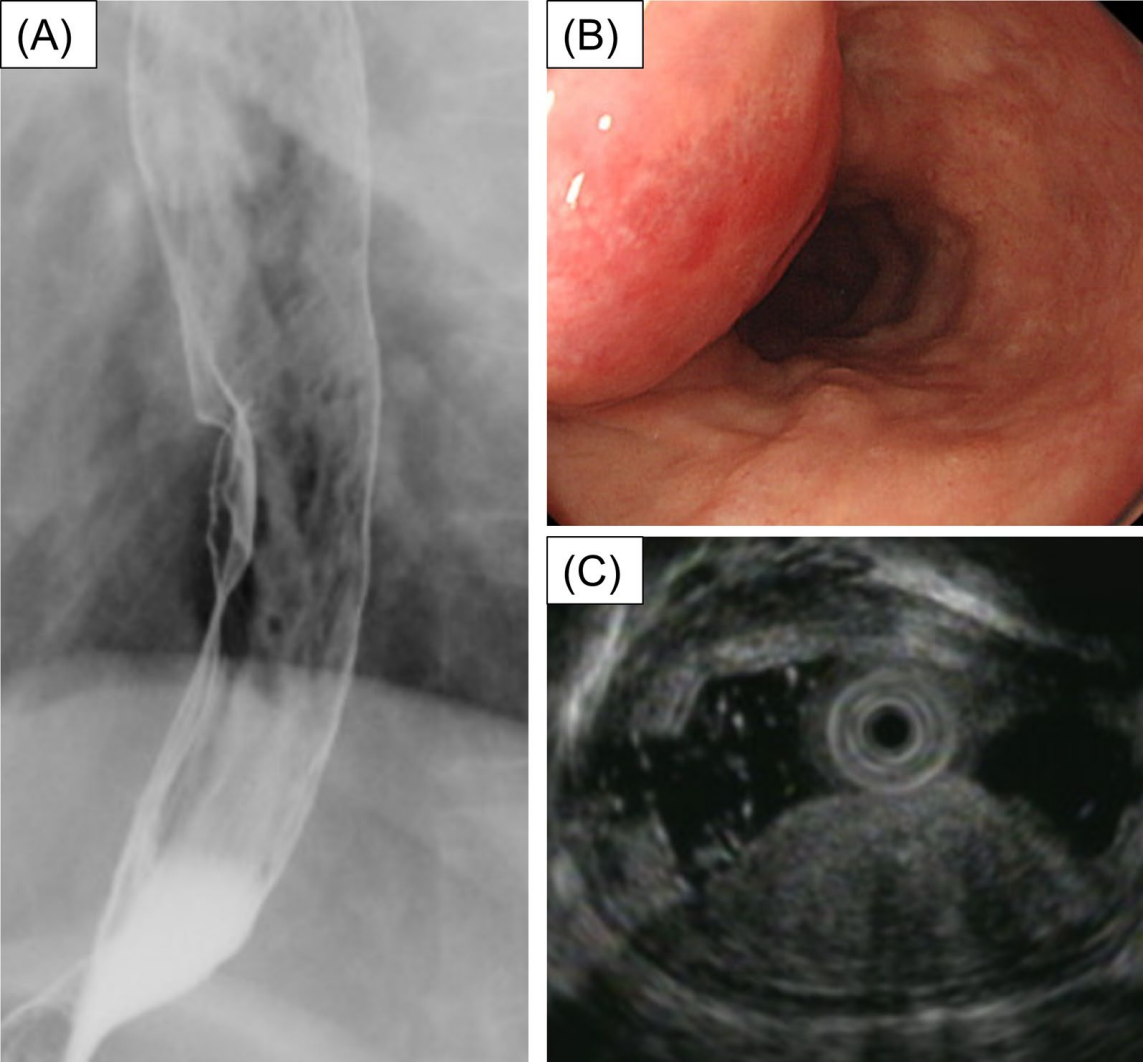
Esophagogram test showed a dome-shaped tumor in lower thoracic esophagus (**A**). Endoscopy showed a round lesion with smooth surface and central redness (**B**). EUS showed a hypoechoic tumor in the second layer (**C**)

**Fig. 2:**
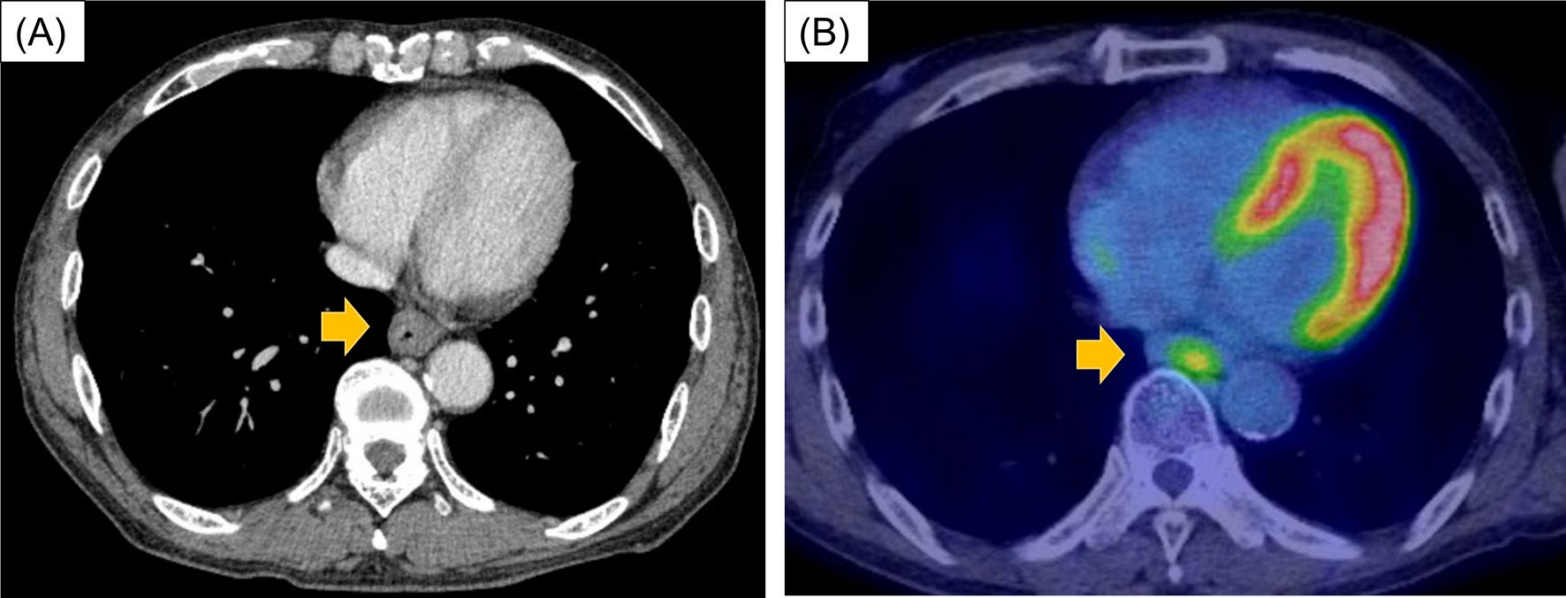
CT showed the tumor as thickness of esophageal wall (**A**). PET showed fluorodeoxyglucose concentration in the esophageal tumor (**B**)

**Fig. 3:**
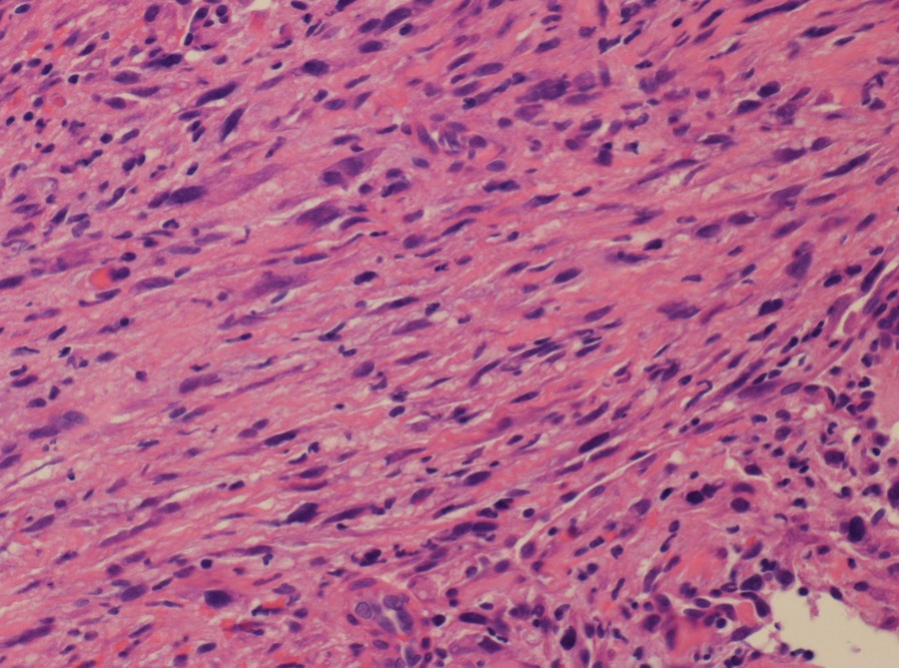
Microscopic examination of the biopsy specimen showed pleomorphic atypical spindle cells with increased mitotic activities. (hemotoxylin and eosin stain, 200 ×)

The patient had no symptoms on admission, and physical examination showed no abnormality except for the operation scar on the abdomen. The results of laboratory tests were normal, and no tumor markers (CEA, CA19-9, SCC, and CYFRA) were elevated. Esophagogram showed a dome-shaped tumor, 3 cm in size, in the lower thoracic esophagus (Fig. 1A). Endoscopy showed a round lesion with a smooth surface and central redness, similar to a submucosal tumor (SMT), located 40 cm from the dental arch (Fig. 1B). Endoscopic ultrasound (EUS) revealed a hypoechoic region in the second layer (Fig. 1C). Contrast computed tomography (CT) showed thickness of the esophageal wall (Fig. 2A) but no findings of metastasis. Positron emission tomography (PET) also showed fluorodeoxyglucose concentration in the esophageal tumor (Fig. 2B) but there was no sign of metastasis in the whole body. Magnetic resonance imaging did not show any specific finding. On boring biopsy, pleomorphic atypical spindle cells with increased mitotic activity under the squamous epithelium were observed on histopathological examination (Fig. 3). Immunohistochemical staining was negative for AE1/AE3, CD117, CD34, S100, desmin, and caldesmon; focally positive for αSMA, cdk4, and Ki-67; and positive for CD68 and p53. Histological examination suggested features of sarcoma; however, a definitive diagnosis was not made.

**Fig. 4:**
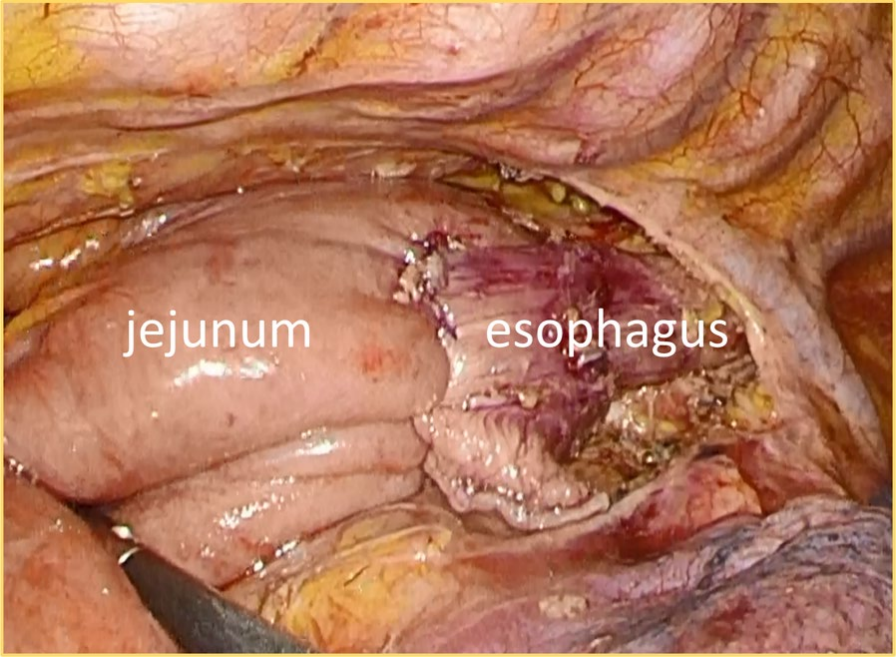
Esophagojejunostomy using overlap technique by thoracoscopic approach

Because the efficacy of chemotherapy or radiotherapy for esophageal sarcoma is unclear and a treatment strategy has not been established, we planned to treat the tumor surgically without neoadjuvant therapy. The patient underwent lower esophagectomy and remnant gastrectomy with jejunal reconstruction. First, laparotomy was done in the supine position, because adhesions from previous surgeries were expected. In fact, there were severe adhesions around the anastomosis sites of the transverse colon, remnant stomach, pancreas, and liver. After dissection of the adhesions, remnant gastrectomy was performed, and the jejunum flap was elevated into the right thoracic cavity through the esophageal hiatus. The abdomen was temporarily closed. The patient was then placed in the prone position and lower esophagectomy and esophagojejunostomy with Roux-en Y reconstruction using the overlap technique was performed under the thoracoscopic approach (Fig. 4). Next, the abdomen was reopened in the supine position and the elevated jejunum was fixed to the esophageal hiatus. Finally, Braun anastomosis was performed and the surgery was completed. Lymph node dissection was not done.

**Fig. 5:**
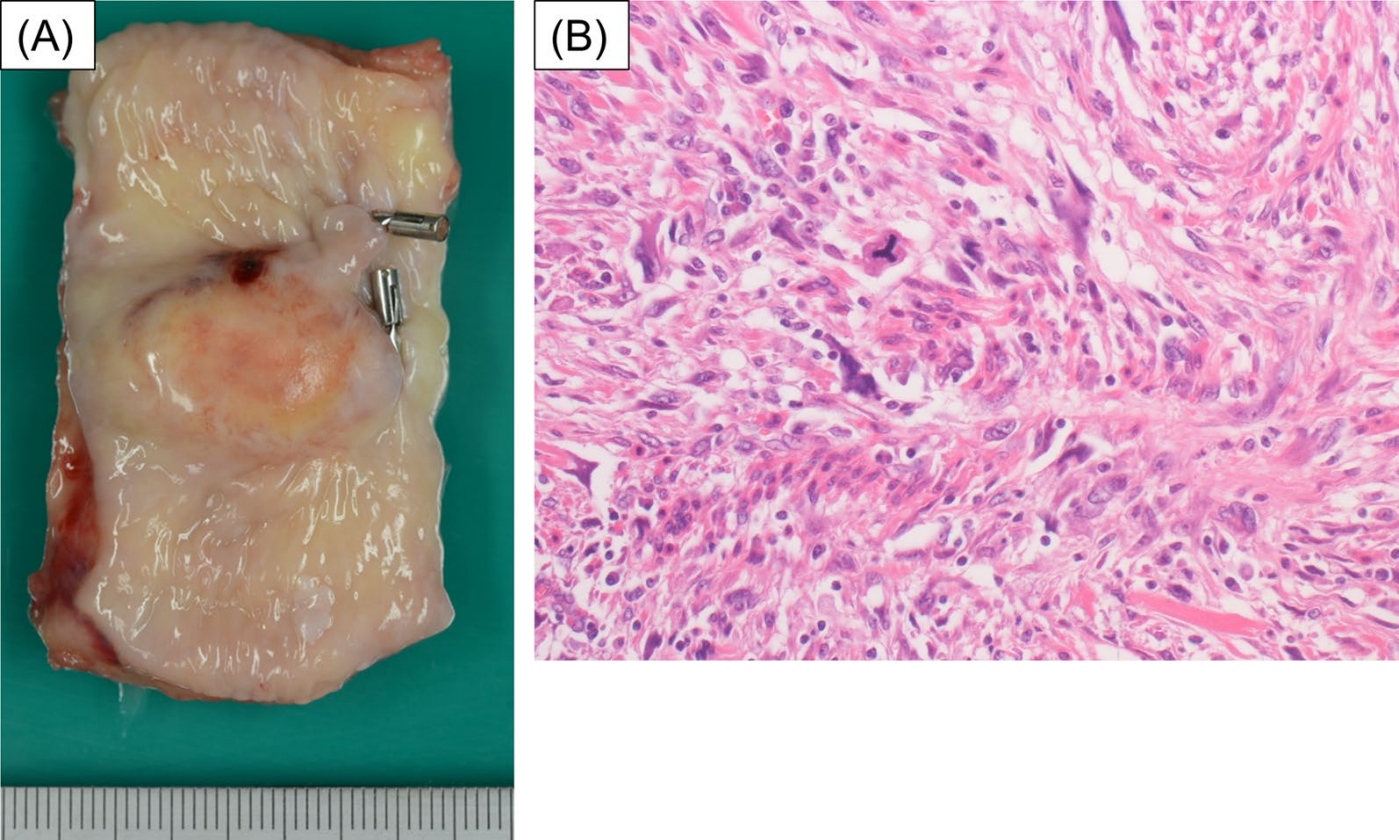
Macroscopic finding of the esophageal tumor, 3.0 × 1.8 cm in size (**A**). Microscopic examination showed the pleomorphic spindle cells proliferation with storiform pattern in the submucosal layer (**B**). (Hemotoxylin and eosin stain, 200 ×)

The surgical specimen contained the tumor SMT, 3.0 × 1.8 cm in size (Fig. 5A). Histopathological examination of the resected tissue revealed pleomorphic spindle cell proliferation with a storiform pattern in the submucosal layer (Fig. 5B). Immunohistochemical staining was negative for AE1/AE3, CD34, S100, desmin, ALK, and ERG; focal positive for caldesmon, SMA, and Ki-67; and positive for CD68, p53, and H3K27me3. Pathological morphology and immunostaining examination resulted in the diagnosis of UPS, excluding other diseases.

The patient did not have any severe postoperative adverse events except abdominal drain infection and was discharged in stable condition approximately 1 month after the surgery. Adjuvant therapy was not administered, and the patient survived without local recurrence or distant metastasis 1 year after surgery.

## Discussion

The UPS was reclassified from MFH according to the WHO classification in 2002, and the name of MFH disappeared in 2013 [3]. The current category of UPS was considered synonymous with the previous MFH [4]. UPS is the second most common type of soft tissue sarcoma [1]. Previous reports demonstrated that UPS tends to occur in extremities (55%), trunk (35%), retroperitoneum (9%), and left atrium (1%), thus UPSE is extremely rare [2]. The characteristics of UPSE in our case and 9 previous reports [5–13] are demonstrated in Table 1. All patients were male and over 40 years of age. Two cases (Our case and Case 7) have cancer history but there were no evidence of tumor recurrence or metastasis when the UPSE was found. Cancer history has not been reported to be a risk factor for UPS. The clinical symptoms of UPSE are nonspecific [11, 12]. All patients, excluding our patient, complained of dysphagia. Tumor markers were measured in three cases (Cases 6 and 8), and were not elevated in any case. Endoscopy is useful for detecting UPSE, and biopsy is essential to rule out other diseases, such as carcinoma. However, a definitive diagnosis of UPS is sometimes difficult by biopsy alone. In our case, a tumor-like SMT was detected on endoscopy. The boring biopsy did not provide a definitive diagnosis; however, sarcoma was suspected.

**Table 1:** Characteristics for undifferentiated pleomorphic sarcoma in esophagus

Case no.	Age (year)/sex	Symptom	Location	Biopsy	Surgery	Appearance	Size (cm)	Depth	Pathology	Prognosis (month)/cause of death
Our case	73/M	None	Lt	Sarcoma	Partial esophagectomy	Submucosal	3	S	UPS	Alive (12)
1 [5]	65/M	Dysphagia	Mt	MFH	Polypectomy	Polypoid	3.3	Unknown	MFH	Alive (3)
2 [6]	59/M	Dysphagia, weight loss, fever	Mt	Malignant tumor	Subtotal esophagectomy	Pedunculated polypoid	6.5	Unknown	MFH	Unknown
3 [7]	46/M	Dysphagia	Mt	Necrosis tissue	Subtotal esophagectomy	Pedunculated polypoid	14	M	MFH + SCC	Unknown
4 [8]	67/M	Dysphagia	Lt	Undifferentiated carcinoma	Esophagogastrectomy, pneumonectomy, jejunum resection	Polypoid	12	A	MFH	Dead (1) /Peritonitis
5 [9]	57/M	Dysphagia, odynophagia, hoarseness	Mt	Negative for carcinoma	Esophagotomy, mucosal resection	Pedunculated polypoid	6	Unknown	MFH	Alive (60)
6 [10]	50/M	Dysphagia, cough, weight loss	Ce	Sarcoma	Total esophagolaryngectomy, tracheostomy	Polypoid	12	S	UPS	Dead (7) /Metastasis
7 [11]	66/M	Dysphagia	Mt	Malignant tumor	Polypectomy	Pedunculated polypoid	4.5	M	MFH + SCC	Unknown
8 [12]	43/M	Dysphagia, odynophagia, weight loss	Ce–Ut	UPS	Total esophagolaryngectomy	Polypoid	7	S	UPS	Alive (12)
9 [13]	78/M	Dysphagia	Mt	Pleomorphic malignant feature	Total esophagectomy	Pedunculated polypoid	7	S	MFH	Alive (12)

*Ce* cervical esophagus, *Ut* upper thoracic esophagus, *Mt* middle thoracic esophagus, *Lt* lower thoracic esophagus, *M* muscularis mucosa, *S* submucosa, *A* Adventia, *MFH* malignant fibrous histiocytoma, *UPS* undifferentiated pleomorphic sarcoma, *SCC* squamous cell carcinoma

The standard treatment for UPS is complete resection, and a wide margin is required, if feasible [1, 10]. A previous retrospective study of 266 cases of UPS of the extremities and trunk demonstrated that patients with inadequate margins had higher fatality rates than those with adequate wide margins [2]. However, in the case of intra-abdominal or gastrointestinal origin tumor, wide margins may be difficult to achieve to preserve organ's function, and NCCN guidelines also suggest that complete resection with negative margins is necessary [14]. All previous UPSE cases were treated by tumor resection, but the techniques varied from esophagectomy to polypectomy (Table 1). Six patients underwent lymph node dissection with esophagectomy, but only one patient with liver metastasis and invasion to other organs (left main bronchus, lung, and atrium) had lymph node metastasis in the small intestinal mesentery (case 4). In our case, surgical resection without lymph node dissection was selected, because the tumor was judged to be completely resectable with negative margins on additional examinations. Because the patient had a history of gastrectomy, transverse colectomy, and radiotherapy to the neck area, we chose partial esophagectomy and reconstruction using the jejunum. Manipulation and anastomosis in the neck area are considered risky. In addition, the residual colon was short, and the middle colonic artery had been cut; therefore, reconstruction using the colon was difficult. Therefore, laparotomy was done first to evaluate how remnant gastrectomy and reconstruction could be undertaken. After the abdominal approach, esophagojejunal anastomosis was performed under thoracoscopy without any adverse event.

Chemotherapy for soft tissue sarcoma can be considered when the tumor is advanced or unresectable. In a previous study on soft tissue sarcoma, chemotherapy with epirubicin and ifosfamide improved both overall and disease-free survival [15]. Moreover, this regimen was superior to gemcitabine plus docetaxel in disease-free survival for high-grade soft tissue sarcoma, including UPS, of the trunk and extremities [16]. Furthermore, clinical studies of immune checkpoint inhibitor regimens such as pembrolizumab, nivolumab, and ipilimumab are being investigated [17, 18]. As mentioned above, chemotherapy for UPS may be useful in the future, but the optimal regimen remains unclear. Radiation therapy for sarcomas has been suggested to have some efficacy. Postoperative radiotherapy is recommended for soft tissue sarcomas with close surgical margins or microscopically positive margins and tumors involving the bone, main vessels, and nerves [19, 20]. Moreover, for UPS of the trunk and extremities, adjuvant radiotherapy has been reported to reduce mortality and occurrence of metastasis [2]. There is no evidence of any effect of chemotherapy or radiotherapy on UPSE. One patient was administered neoadjuvant chemotherapy (mesna, doxorubicin, ifosfamide, and dacarbazine), and the tumor size reduced slightly (Case 8). Another patient experienced local recurrence and metastasis 1 month after surgery, and chemotherapy (cisplatin, ifosfamide, and doxorubicin) was administered. However, the region of local recurrence expanded and metastasis worsened (case 6). In our case, postoperative adjuvant therapy was not prescribed after adequate discussion among multidisciplinary professionals and family members.

The prognosis for UPS remains poor. The rate of local recurrence after surgery was 15%, and that of metastasis was 37.6% in UPS with inadequate surgical margins [10]. Among the 10 UPSE cases, only 4 survived over 12 months after surgery (our case and cases 5, 8, and 9). However, a standard treatment for UPSE has not been established yet; therefore, further knowledge of UPSE is required.

## Conclusions

We report a rare case of UPSE, that was successfully treated with surgical resection. Currently, complete resection is the only treatment option for UPSE. An optimal treatment strategy using chemotherapy or radiotherapy should be established.

## Data Availability

Not applicable.

## Abbreviations

UPS: Undifferentiated pleomorphic sarcoma
MFH: Malignant fibrous histiocytoma
UPSE: Undifferentiated pleomorphic sarcoma in the esophagus
ESD: Endoscopic submucosal dissection
SMT: Submucosal tumor
EUS: Endoscopic ultrasound
CT: Computed tomography
PET: Position emission tomography
